# Russeting in Apple is Initiated after Exposure to Moisture Ends: Molecular and Biochemical Evidence

**DOI:** 10.3390/plants10010065

**Published:** 2020-12-30

**Authors:** Jannis Straube, Yun-Hao Chen, Bishnu P. Khanal, Alain Shumbusho, Viktoria Zeisler-Diehl, Kiran Suresh, Lukas Schreiber, Moritz Knoche, Thomas Debener

**Affiliations:** 1 Institute of Plant Genetics, Molecular Plant Breeding Section, Leibniz University Hannover, Herrenhäuser Straße 2, 30419 Hannover, Germany; straube@genetik.uni-hannover.de; 2 Institute of Horticultural Production Systems, Fruit Science Section, Leibniz University Hannover, Herrenhäuser Straße 2, 30419 Hannover, Germany; chen@obst.uni-hannover.de (Y.-H.C.); khanal@obst.uni-hannover.de (B.P.K.); shumbusho1@uniba.sk (A.S.); moritz.knoche@obst.uni-hannover.de (M.K.); 3 Institute of Cellular and Molecular Botany (IZMB), Department of Ecophysiology, University of Bonn, Kirschallee 1, 53115 Bonn, Germany; vzeisler@uni-bonn.de (V.Z.-D.); s6kisure@uni-bonn.de (K.S.); lukas.schreiber@uni-bonn.de (L.S.)

**Keywords:** russet, cuticle, periderm, *Malus × domestica*, cutin, wax, suberin

## Abstract

Exposure of the fruit surface to moisture during early development is causal in russeting of apple (*Malus* × *domestica* Borkh.). Moisture exposure results in formation of microcracks and decreased cuticle thickness. Periderm differentiation begins in the hypodermis, but only after discontinuation of moisture exposure. Expressions of selected genes involved in cutin, wax and suberin synthesis were quantified, as were the wax, cutin and suberin compositions. Experiments were conducted in two phases. In Phase I (31 days after full bloom) the fruit surface was exposed to moisture for 6 or 12 d. Phase II was after moisture exposure had been discontinued. Unexposed areas on the same fruit served as unexposed controls. During Phase I, cutin and wax synthesis genes were down-regulated only in the moisture-exposed patches. During Phase II, suberin synthesis genes were up-regulated only in the moisture-exposed patches. The expressions of cutin and wax genes in the moisture-exposed patches increased slightly during Phase II, but the levels of expression were much lower than in the control patches. Amounts and compositions of cutin, wax and suberin were consistent with the gene expressions. Thus, moisture-induced russet is a two-step process: moisture exposure reduces cutin and wax synthesis, moisture removal triggers suberin synthesis.

## 1. Introduction

Russeting is a surface disorder of many fruitcrop species including of apple [1,2,3,4,5]. Russeting is characterized by the formation of rough, brownish patches on the fruit skin. The impaired appearance of the skin reduces the fruit’s marketability and an associated increase in water vapor permeability compromises its postharvest performance [6]. In botanical terms, russet is the result of the formation of a periderm, the cell walls of the phellem being suberized. The periderm assumes the barrier functions of the epidermis and cuticle—the fractured epidermal cells soon drying and sloughing off. Despite of its economic importance, the sequence of processes that lead to russeting are not entirely clear.

Some progress has been made in genetic analyses. Using crosses of apple clones that differ in russet susceptibility Falginella [7] and Lashbrooke [8] identified several QTL (Quantitative Trait Locus) regions on chromosomes 2, 12 and 15 that affected russet susceptibility under field conditions [7,8]. Within these, SHN3 was located and identified as a candidate gene responsible for fruit skin development due to its differential expression in russeted and non-russeted clones [8]. Legay [9] compared gene expressions in russeted and non-russeted mature fruit of a range of apple cultivars. A number of differentially-regulated genes were identified. Some of these were related to cutin, wax, suberin and lignin synthesis and others to the transport and transcriptional regulation of these moieties [9]. Unfortunately, all these studies focused on fruits at the mature stage. The only exception was Lashbrooke [8] who also investigated an early green stage. To our knowledge, there is no further information available on russeting during early fruit development of apple—when russeting susceptibility is at maximum [1,10,11,12,13,14]. Further, comparison of differential gene expressions in fruits from russeted and non-russeted genotypes may not be conclusive, since properties other than russet susceptibility may also differ.

Microscopic cracks (‘microcracks’) in the cuticle are the first visible symptom of russeting [10,15,16]. Microcracks form when the fruit skin is strained during periods of rapid surface expansion [17]. This period typically occurs during early fruit development [1,10,11,12,13,14,17]. Microcracking is exacerbated by surface moisture [18,19,20]. Recently, a system was developed that allows microcracks, and hence also russet, to be induced in the skins of developing apples by localized exposure to moisture [15]. The remaining unexposed skin of the same fruit may serve as the control. Briefly, a short length of tube is mounted on the fruit surface and filled with water. This procedure exposes a defined patch of the fruit surface to water, while the remaining fruit surface represents the unexposed control. A periderm forms in the skin area defined by the tube aperture due to the induction of microcracks by moisture. This experimental setup avoids a number of shortcomings associated with comparisons of fruits of different genotypes or fruits of the same genotype but collected from different sites, from different trees or even from different positions in the canopy of the same tree. It thus allows critical comparisons to be made by eliminating a range of potential sources of variability in russet formation, such as by the stage of fruit development, the microenvironment of the fruit in the canopy, etc. Using this system, the effect of moisture exposure on the histology of russet formation was investigated in greater detail [16]. Several findings were reported: (1) Microcracking of the cuticle occurred during moisture exposure, but there was no periderm formation during moisture exposure. (2) Cuticle deposition ceased during moisture exposure. (3) After removal of the moisture treatment a periderm formed within 4 d, regardless of the duration of moisture exposure. (4) The periderm formed in the hypodermis, several cell layers beneath a microcrack. (5) There was no difference in histology between natural and artificial moisture-induced russet. Unfortunately, the time resolution of such histological studies is limited. Moreover, changes at the transcriptional and biochemical levels will precede those detected at the histological level.

To develop a better understanding of the mechanism(s) of russet formation we (1) investigated the expressions of genes putatively involved in cutin, wax and suberin synthesis and (2) analyzed the compositions of the cuticle and the periderm during and after moisture exposure. To identify whether duration of moisture exposure was a factor in russeting, the fruit skin was exposed to continuous surface moisture for 6 or for 12 d periods. We focused on those genes that were found to be differentially expressed in russeted and non-russeted apple in previous studies [8,9,21].

## 2. Results

### 2.1. Changes in Gene Expression and Metabolism in Young Fruit During and after Moisture Exposure

**Figure 1:**
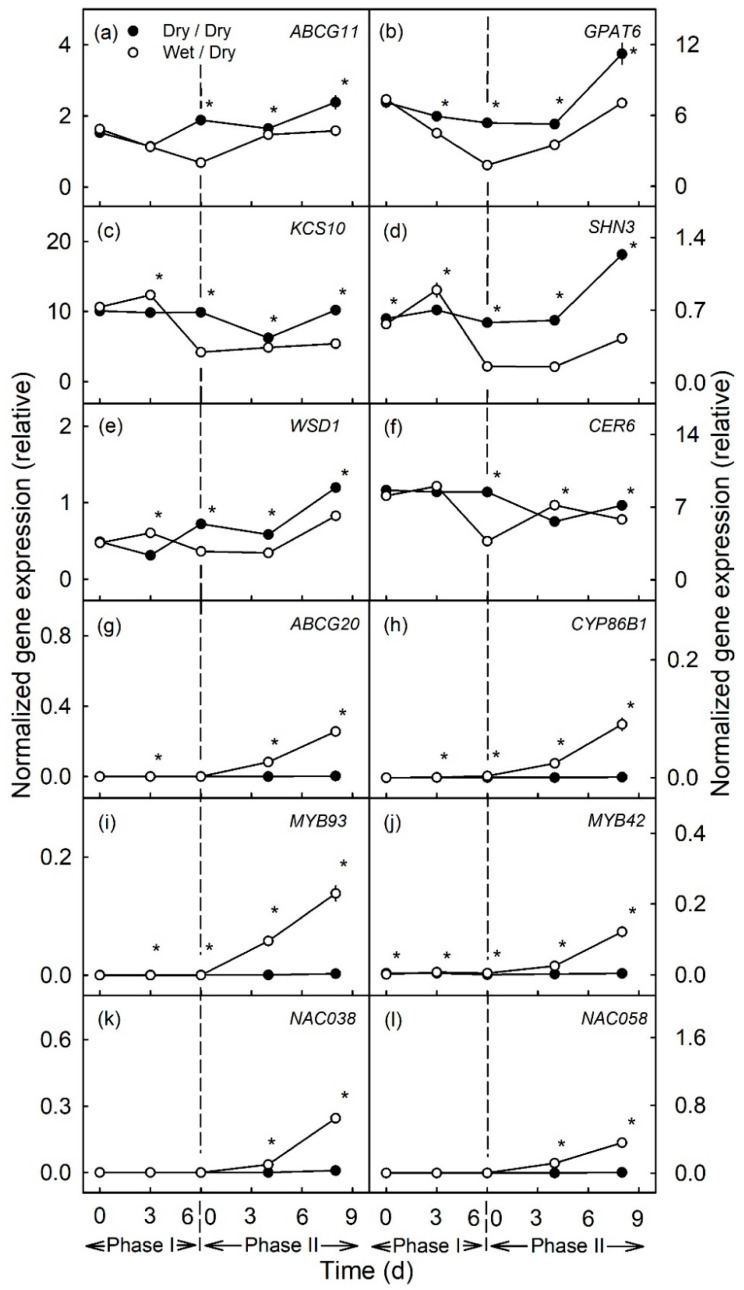
Time courses of expression of genes related to cutin and wax synthesis (**a**–**f**) and to suberin and lignin synthesis (**g**–**l**) of apple fruit skin during (Phase I) of exposure to moisture and after exposure was discontinued (Phase II). During Phase I, a patch of the fruit skin was exposed to moisture for 6 d beginning at 31 days after full bloom (DAFB) (wet). During the subsequent Phase II, moisture was removed, and the patch was exposed to the atmosphere (dry). Moisture-exposed patches of the fruit skin are referred to as wet/dry, unexposed control patches as dry/dry. The end of moisture exposure is indicated by the vertical dashed line. The expression values are means ± SE of three independent biological replicates comprising ten fruit each. The ‘*’ indicates significant differences between dry/dry and wet/dry at *p* ≤ 0.05 (Student’s *t*-test).

**Figure 2:**
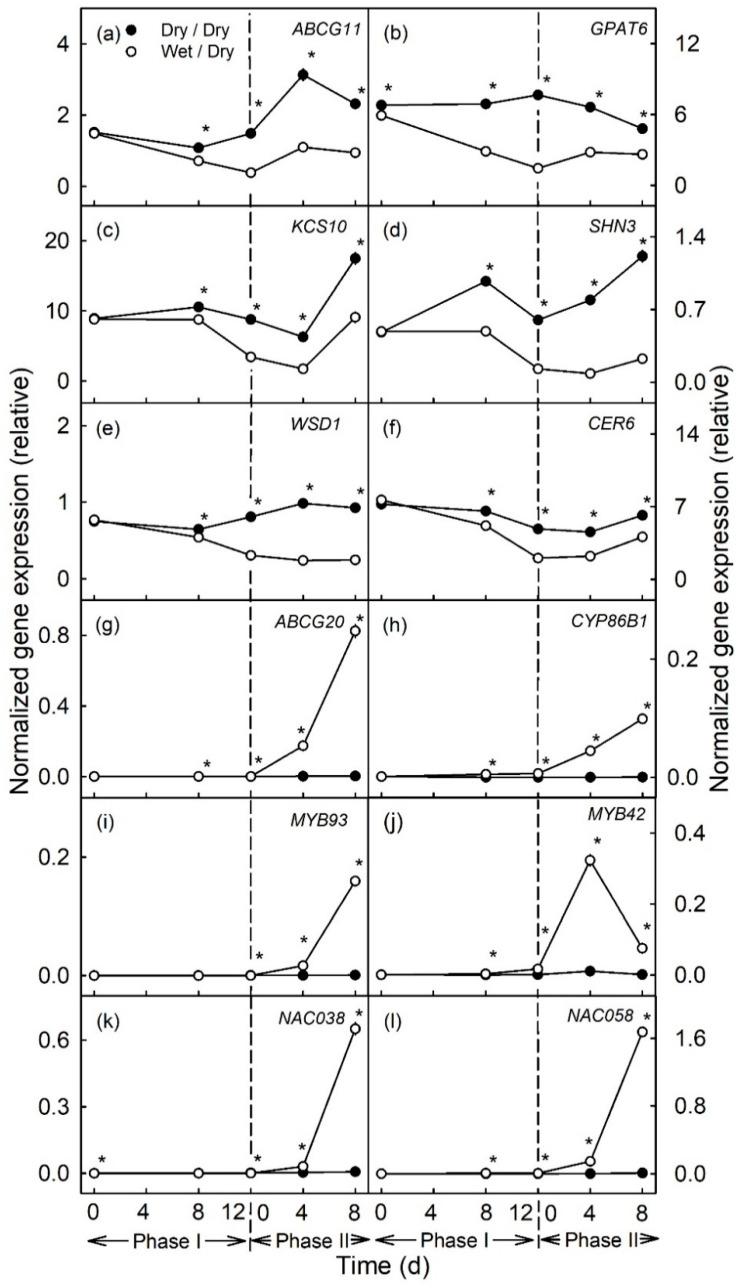
Time courses of expression of genes related to cutin and wax synthesis (**a**–**f**) and to suberin and lignin synthesis (**g**–**l**) of apple fruit skin during (Phase I) of exposure to moisture and after exposure to moisture was discontinued (Phase II). During Phase I, a patch of the fruit skin was exposed to moisture for 12 d beginning at 31 days after full bloom (DAFB) (wet). During the subsequent Phase II, moisture was removed, and the patch was exposed to the atmosphere (dry). Moisture-exposed patches of the fruit skin are referred to as wet/dry, unexposed control patches as dry/dry. The end of moisture exposure is indicated by the vertical dashed line. The expression values are means ± SE of three independent biological replicates comprising ten fruit each. The ‘*’ indicates significant differences between dry/dry and wet/dry at *p* ≤ 0.05 (Student’s *t*-test).

During moisture exposure (Phase I) beginning at 31 days after full bloom (DAFB), genes involved in cutin (*ABCG11*, *GPAT6*) and wax (*KCS10*, *SHN3*, *WSD1* and *CER6*) syntheses were significantly down-regulated compared to in the un-exposed (dry) control (Figure 1a–f and Figure 2a–f). The down-regulation occurred fairly consistently for all genes and after both the 6 d and the 12d moisture exposure (Figure 1a–f and Figure 2a–f). The longer exposure duration generally resulted in a greater down-regulation. The down-regulation was consistent for *ABCG11*, *GPAT6*, *KCS10* and *SHN3* in all three seasons of the experiment but down-regulation was less for *WSD1* and *CER6*, particularly in the 2018 season (Figures S1a–f and S2a–f).

In contrast, there was no change in expression of genes related to suberin synthesis (*ABCG20*, *CYP86B1*, *MYB93*) during moisture exposure (Phase I) (Figure 1g–i and Figure 2g–i). *MYB42*, a regulator of lignin synthesis, was slightly but significantly up-regulated during moisture exposure (Figure 1j and Figure 2j). Meanwhile, *NAC038* and *NAC058*, that do not yet have assigned functions, were not differentially expressed during Phase I (Figure 1k–l and Figure 2k–l).

After discontinuation of moisture exposure (Phase II), the expression of cutin- and wax-related genes in the moisture-exposed patches increased again slightly but the relative expressions were still significantly lower than the expressions of these genes in the control patches of the same fruit. The relative expression of *CER6* in the 6 d moisture treatment, was generally similar in the moisture-exposed and control patches (Figure 1a–f and Figure 2a–f).

In contrast, suberin- and lignin-related genes were consistently up-regulated, regardless of whether the moisture exposure during Phase I was for 6 or for 12 d (Figure 1g–l and Figure 2g–l, Figures S1g–l and S2g–l). The up-regulation of expression increased from 4 to 8 d after discontinuation of moisture exposure. Only for *MYB42* was a transient peak in expression observed at 4 d after moisture exposure (Figure 2j).

### 2.2. Changes in Gene Expression and Metabolism Caused by Moisture Exposure (Phases I and II) during Later Stages of Fruit Development

**Figure 3:**
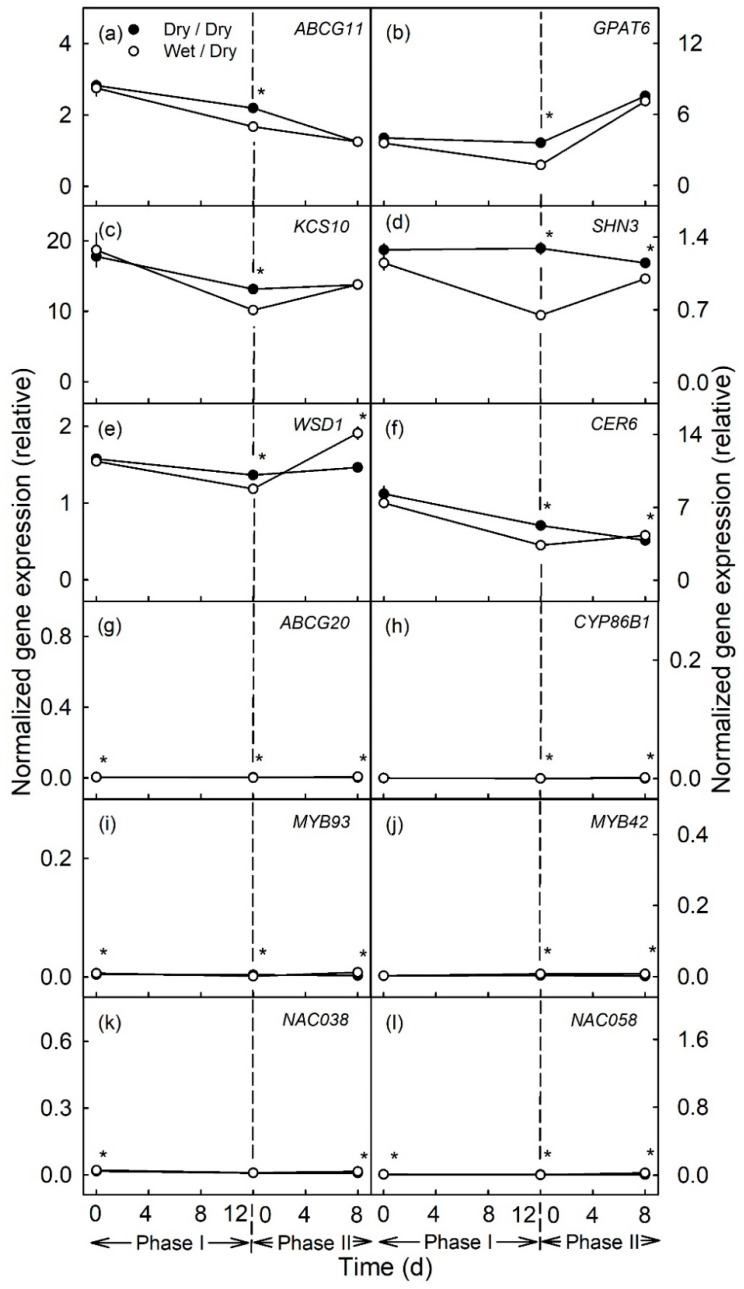
Time course of expression of genes related to cutin and wax synthesis (**a**–**f**) and to suberin and lignin synthesis (**g**–**l**) of apple fruit skin during moisture exposure (Phase I) and after exposure to moisture was discontinued (Phase II). During Phase I, a patch of the fruit skin was exposed to moisture for 12 d beginning at 66 days after full bloom (DAFB) (wet). During the subsequent Phase II, moisture was removed, and the patch was exposed to the atmosphere (dry). Moisture-exposed patches of the fruit skin are referred to as wet/dry, unexposed control patches as dry/dry. The end of moisture exposure is indicated by the vertical dashed line. The expression values are means ± SE of three to five independent biological replicates comprising ten fruit each. The ‘*’ indicates significant differences between dry/dry and wet/dry at *p* ≤ 0.05 (Student’s *t*-test).

**Figure 4:**
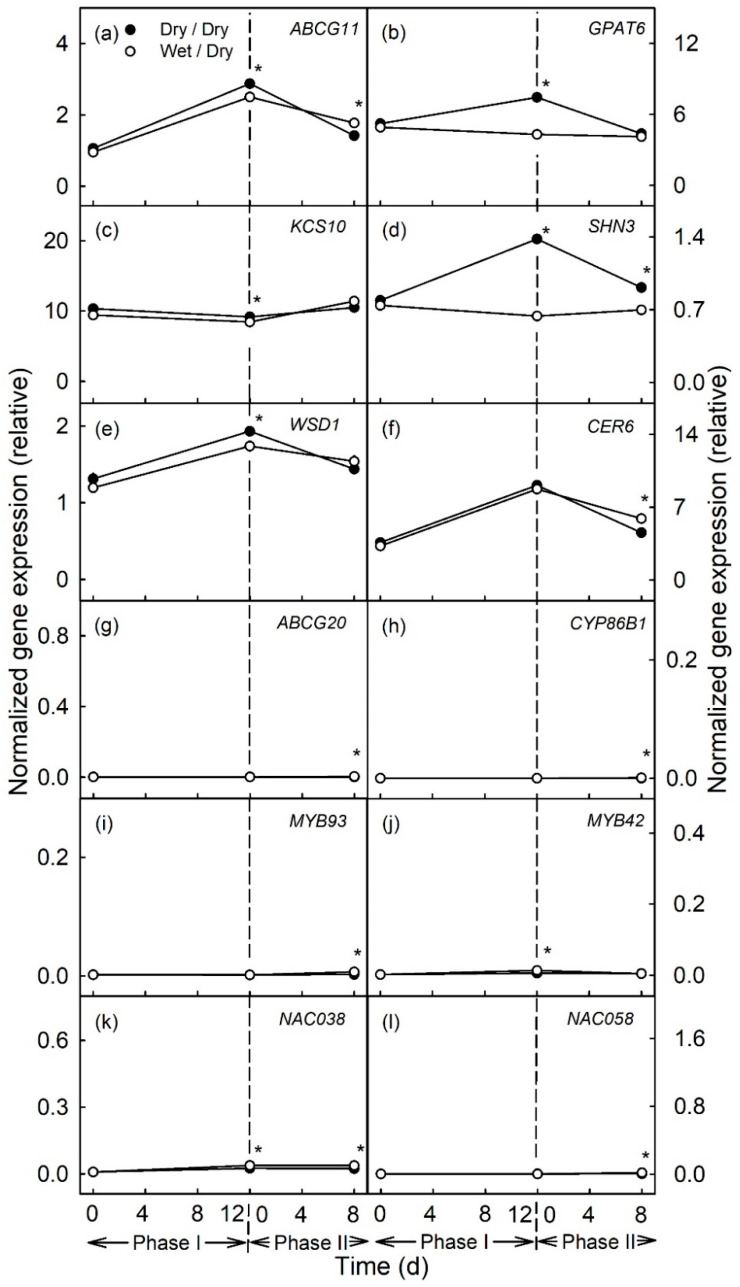
Time course of expression of genes related to cutin and wax synthesis (**a**–**f**) and to suberin and lignin synthesis (**g**–**l**) of apple fruit skin during exposure to moisture (Phase I) and after exposure to moisture was discontinued (Phase II). During Phase I, a patch of the fruit skin was exposed to moisture for 12 d beginning at 93 days after full bloom (DAFB) (wet). During the subsequent Phase II, moisture was removed, and the patch was exposed to the atmosphere (dry). Moisture exposed patches of fruit skin are referred to as wet/dry, unexposed control patches as dry/dry. The end of the moisture exposure is indicated by the vertical dashed line. The expression values are means ± SE of three independent biological replicates comprising ten fruit each. The ‘*’ indicates significant differences between dry/dry and wet/dry at *p* ≤ 0.05 (Student’s *t*-test).

In the later stages of fruit development moisture exposure [from 66–78 DAFB (Figure 3a–f) and from 93–105 DAFB (Figure 4a–f)] also caused the down-regulation of the genes related to cutin and wax synthesis, as compared to the unexposed controls. However, the magnitudes of the down-regulations of expression were markedly less than for moisture exposure during the early stages of fruit development (moisture exposure from 31–43 DAFB). There were no changes in expressions of genes related to suberin or lignin synthesis, either during Phase I or Phase II (Figure 3g–l and Figure 4g–l).

### 2.3. Histological and Metabolic Changes during and after Moisture Exposure 

**Figure 5:**
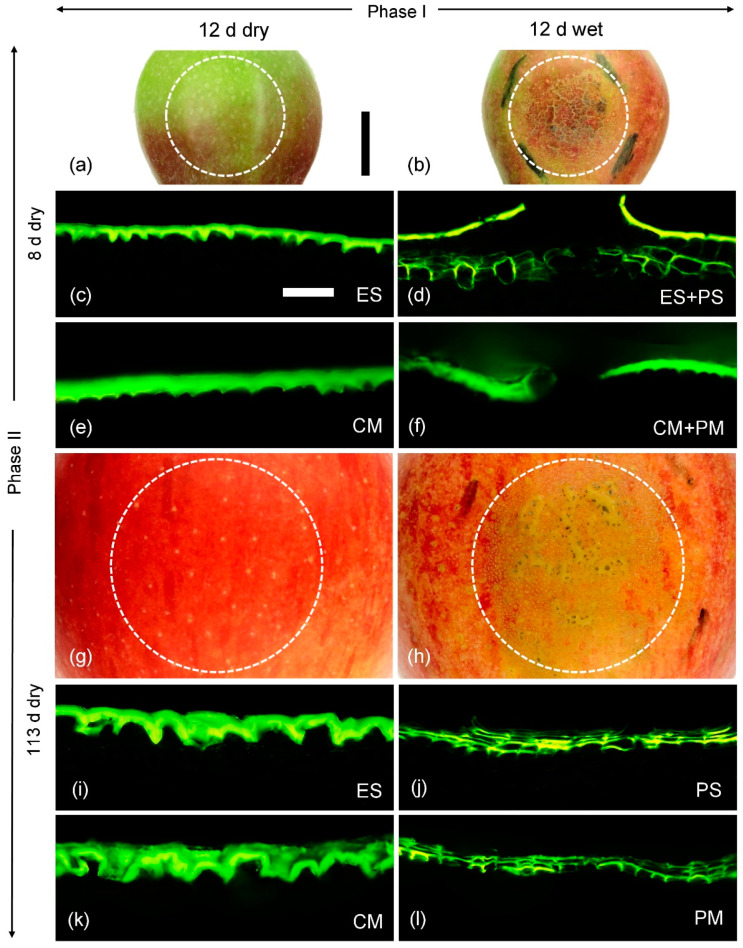
Macroscopic view of unexposed control patches (**a**,**g**) and moisture exposed (**b**,**h**) skin patches of apple fruit. Cross-sections of epidermal skin samples (ES) of control patches (**c**,**i**) and of the composite skins of moisture-exposed patches comprising epidermal plus peridermal sections (ES+PS) (**d**) or peridermal section only (PS) (**j**). Cross-sections of isolated cuticular membranes (CM) (**e**,**k**) and cuticular plus periderm membranes (CM+PM) (**f**) or periderm membranes only (PM) (**l**). The moisture treatment was applied as a two-phase experiment. During Phase I, a patch of the fruit skin was exposed to moisture for 12 d beginning at 31 days after full bloom (DAFB) (wet). During the subsequent Phase II moisture was removed, and the patch was exposed to the atmosphere (dry) (**b**,**d**,**f**,**h**,**j**,**l**). A portion of the unexposed surface on the same fruit served as control (**a**,**c**,**e**,**g**,**i**,**k**). Micrographs were taken 8 d (**a**–**f**) and 113 d (**g**–**l**) after moisture exposure was discontinued. Images in (**c**–**f**) and (**i**–**l**) were taken under incident fluorescent light (U-MWB) after staining with Fluorol Yellow 088. The scale bar in (**a**) equals 10 mm and is representative for all surface views (**a**,**b**,**g**,**h**). The scale bar in (**c**) equals 50 µm and is representative for all cross-sections of the composite (**c**–**f**, **i**–**l**). The dotted circles in (**b**) and (**h**) mark the original footprint of the tube that was mounted on the fruit surface to enable moisture exposure, the dotted circles in (**a**) and (**g**) are unexposed control patches on the same fruit. For details of the moisture treatment, see Materials and Methods.

The skin patches with and without moisture exposure differed in both appearance and composition. The surfaces of skin samples of the unexposed controls comprised a cuticle, occasionally interrupted by lenticels (Figure 5a,g). There was no macroscopically or microscopically detectable periderm, except for that associated with the lenticels (Figure 5c,i). However, for the moisture-exposed skin patches, there were large areas of periderm (Figure 5b,h). A periderm had begun to develop in the underlying hypodermis by 8 d after moisture exposure was discontinued (Figure 5d). By 113 d after discontinuation of moisture exposure, both the periderm thickness and also the proportion of the area covered by periderm within the tube footprint had increased markedly (Figure 5h,j). At this stage, the periderm had reached the fruit surface and was visible macroscopically as irregular, brown patches.

When skin patches were subjected to enzymatic isolation using cellulase and pectinase, the isolated polymers obtained 8 d after moisture exposure had been discontinued in the exposed, and also in the unexposed control patches, comprised only cutin and wax, but no periderm (Figure 5e,f). The periderm that had begun to develop in the hypodermis of moisture-exposed patches and that was also plainly visible in cross-sections under the light microscope (Figure 5d) was probably lost during the isolation process. Thus, it is not surprising that suberin was detectable only in trace amounts in the GC-MS analyses at 8 d after moisture exposure had been discontinued. In contrast, by 113 d after moisture exposure had been discontinued, the periderm in the moisture-exposed patches had extended to the surface and ‘connected’ to the overlying cuticle. This periderm also remained connected during isolation (Figure 5l). There was no detectable periderm in the polymer membrane isolated from the moisture-unexposed (control) patches (Figure 5k).

**Figure 6:**
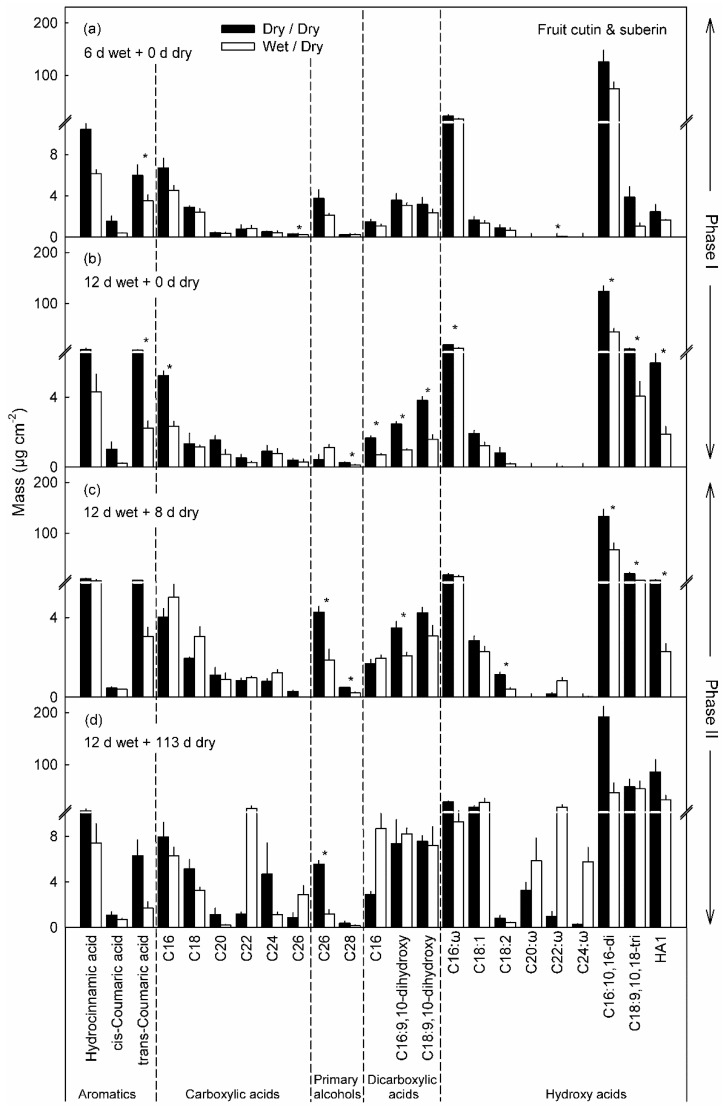
Cutin and suberin monomers in patches of apple fruit skin that were exposed to moisture for 6 d (**a**) and 12 d (**b**) (Phase I, wet). During the subsequent Phase II, the moisture exposure was discontinued (dry) and the cutin and suberin compositions of the patches analyzed after 8 d (**c**) and 113 d (**d**) after moisture exposure was discontinued. Unexposed patches of the fruit skin that remained dry throughout, served as controls (dry/dry). Data represent means ± SE of two to three replicates comprising cuticles of five fruit each. Significance of differences between dry/dry and wet/dry at *p* ≤ 0.05 are indicated by ‘*’ (Student’s *t*-test).

Moisture exposure also altered the cutin and wax compositions. The most abundant constituents of the cutin were the hydroxy fatty acids, i.e., 16-hydroxy-C_16_ acid, 10,16-dihydroxy-C_16_ acid and 9,10,18-trihydroxy-C_18_ acid (Figure 6a). Compared with the unexposed controls, in the cuticles of the moisture-exposed patches these constituents were significantly reduced (Figure 6a,b). Moisture exposure also decreased the levels of trans-coumaric acid, α,ω-dicarboxylic-C_16_ acid, 9,10-dihydroxy-α,ω-dicarboxylic-C_16_ acid and 9,10-dihydroxy-α,ω-dicarboxylic-C_18_ acid. Similarly, the content of carboxylic-C_16_ acid was reduced after 12 d of moisture exposure. The reductions were even more pronounced as the duration of moisture exposure increased from 6 to 12 d. After discontinuation of moisture exposure (Phase II) the amounts of ω-hydroxy-C_20_, -C_22_ and -C_24_ acids and of carboxylic-C_22_ acid in the moisture-exposed patches all increased and were significantly higher than in the unexposed control patches. The amounts of α,ω-dicarboxylic acids, which decreased during Phase I, increased again during Phase II in the moisture-exposed patches (Figure 6c,d).

**Figure 7:**
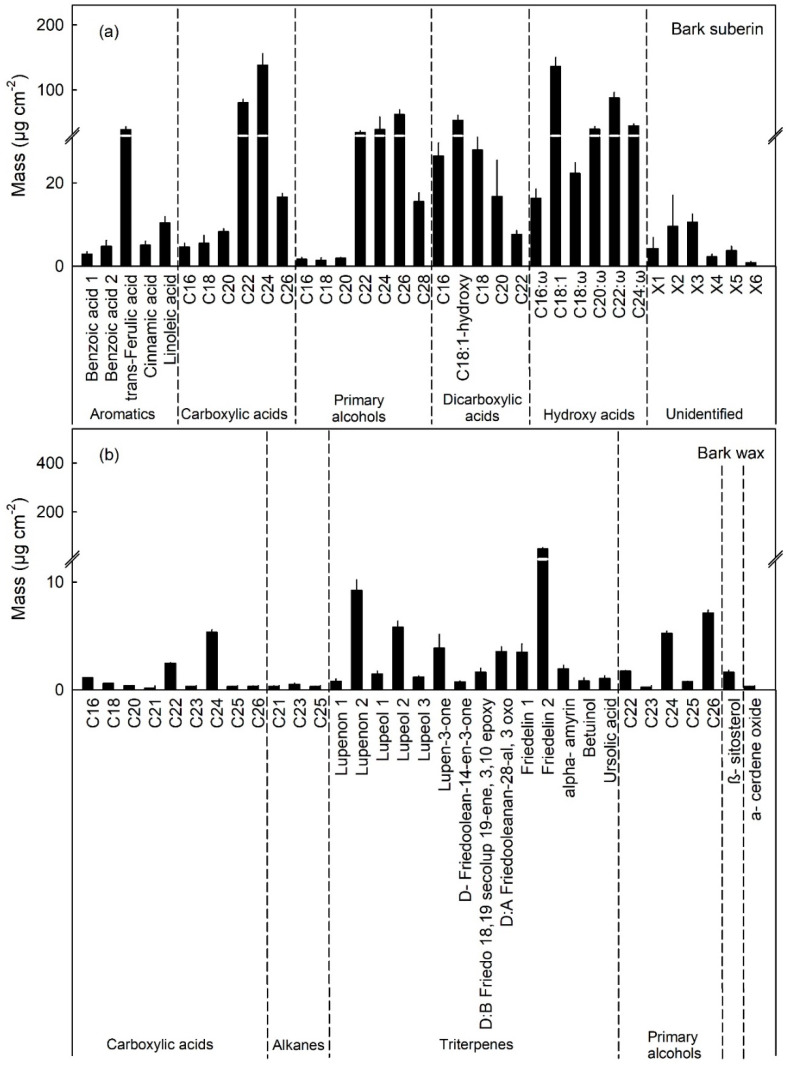
Composition of the periderm of the bark of the trunk (BP) of a ‘Pinova’ apple tree. (**a**) Constituents of the suberin and (**b**) constituents of the wax. The BP represents a pure periderm without any remnants of a cuticle.

The ω-hydroxy-C_20_, -C_22_ and -C_24_ acids are characteristic and unique suberin monomers as indexed by the composition of the pure periderm (i.e., no cuticle) of the bark of the apple tree trunk (Figure 7a). For all other constituents of cutin, and for the wax, there was significant overlap in composition between the cutin and wax of fruit cuticle and of the bark periderm (Figure 7a,b). Normalizing for the three unique characteristic constituents allowed estimation of suberin mass per unit area of the mixed cuticle/periderm composites of the moisture-exposed fruit skin patches.

**Figure 8:**
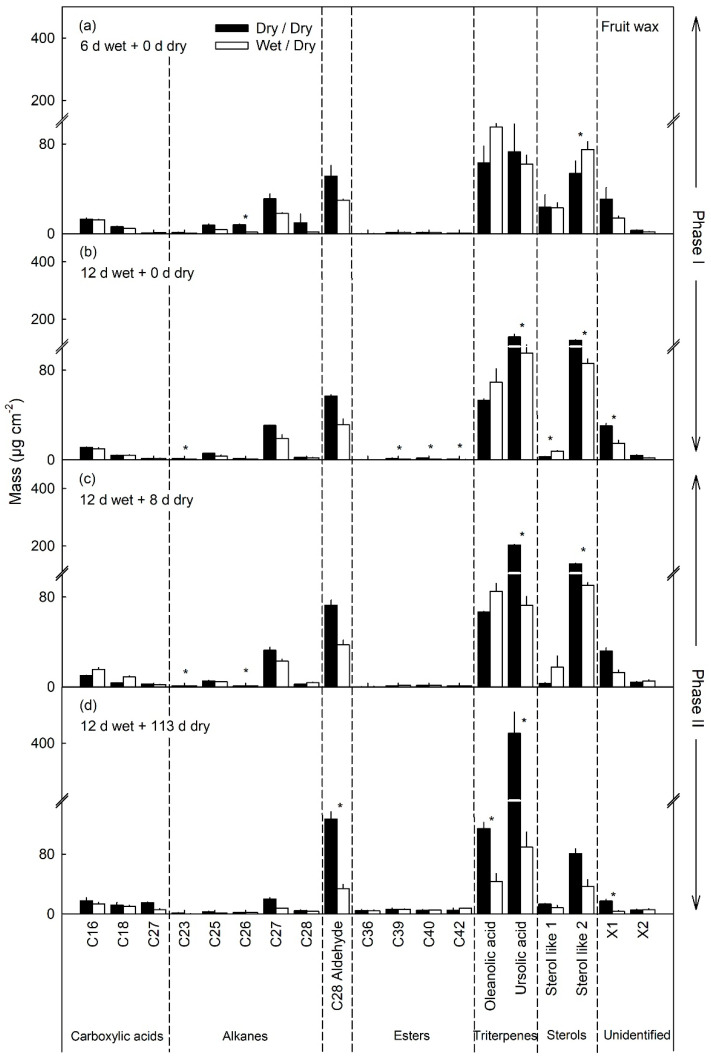
Wax constituents in patches of apple fruit skin that had been exposed to moisture for 6 d (**a**) and for 12 d (**b**) (Phase I, wet). During the subsequent Phase II, the moisture exposure was discontinued (dry) and the cutin and suberin compositions of the patches analyzed after 8 d (**c**) and 113 d (**d**). Unexposed patches of the fruit skin served as controls (dry/dry). Data represent means ± SE of two or three replicates comprising cuticles of five fruit each. Significance of differences between dry/dry and wet/dry at *p* ≤ 0.05 is indicated by ‘*’ (Student’s *t*-test).

The most abundant components of the wax were the triterpenes (oleanolic acid and ursolic acid), the sterols and C_28_ aldehyde. All of these were significantly lower in the moisture-exposed patches compared with the unexposed patches (Figure 8). The mass per area remained constant in the moisture-exposed patches but continued to increase in the unexposed control patches (Figure 8a,b). This pattern was particularly evident for the amounts of ursolic acid and C_28_ aldehyde that increased markedly up to maturity in the unexposed control patches—but not in the moisture-exposed patches (Figure 8).

**Figure 9:**
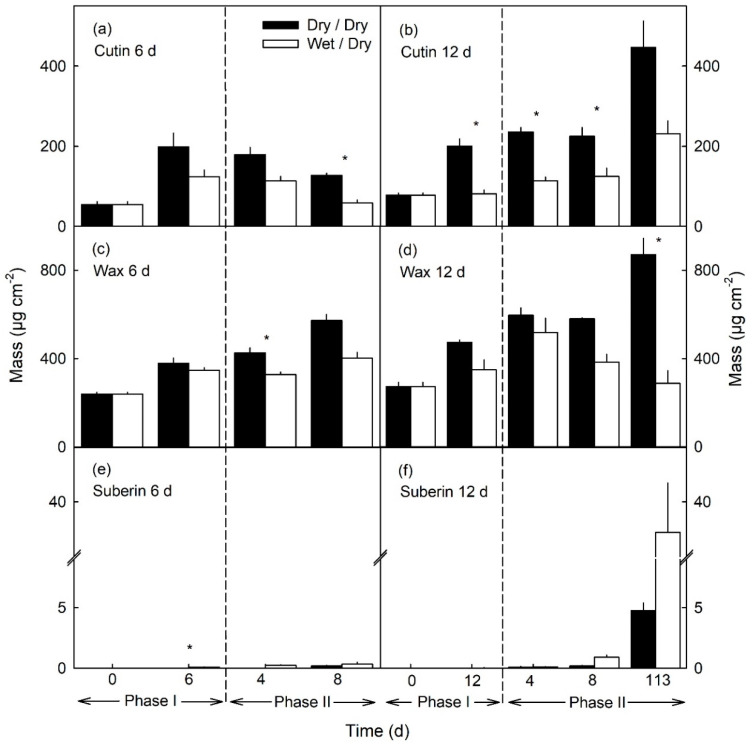
Total mass of cutin (**a**,**b**), wax (**c**,**d**) and suberin (**e**,**f**) in patches of the apple fruit skin during exposure to moisture (Phase I) and after exposure to moisture had been discontinued (Phase II). During Phase I, a patch of the skin was exposed to moisture for 6 d (**a**,**c**,**e**) or 12 d (**b**,**d**,**f**) beginning at 31 days after full bloom (DAFB) (wet). During the subsequent Phase II, the exposure to moisture was discontinued and the patch exposed to the atmosphere (dry). Moisture exposed patches of fruit skin are referred to as wet/dry, unexposed control patches as dry/dry. The end of the moisture exposure period is indicated by the vertical dashed line. The data represent the means ± SE of two or three samples comprising five fruits each. Significance of differences between dry/dry and wet/dry at *p* ≤ 0.05 is indicated by ‘*’ (Student’s *t*-test).

The compositional changes of individual constituents described above resulted in significant changes in the masses per unit area of cutin, wax and suberin. The masses of cutin and wax per unit area were lower in moisture-exposed patches, compared to the unexposed controls (Figure 9a–d). The decreases in mass occurred primarily during Phase I. They remained at about the same levels during the subsequent Phase II until 8 d after moisture exposure had been discontinued. The changes were qualitatively identical for 6 and 12 d of moisture exposure but were larger quantitatively for the longer exposure time (Figure 9a–d). During Phase I, suberin was essentially undetectable, regardless of the duration of moisture exposure. However, low levels of suberin were detectable 8 d after moisture exposure had been discontinued, while levels were markedly higher at 113 d (Figure 9e,f). It is interesting to note that some suberin deposition—albeit at low levels—was also recorded in the unexposed control patches. This last is not surprising because lenticels occur naturally in the unexposed control patches of an apple fruit skin and represent small areas of periderm usually associated with degenerate stomata (Figure 5g) [22].

## 3. Discussion

Our results establish that:

(1) Moisture exposure resulted in down-regulation of the genes involved in cutin and wax synthesis and deposition. The discontinuation of moisture exposure resulted in the up-regulation of genes involved in suberin synthesis.
(2) The early fruit development stage was more responsive to moisture than later stages when effects of moisture exposure on cutin and wax deposition were much less and those on suberin deposition essentially absent.

### 3.1. Gene Expression

Expressions of the genes involved in all steps of cuticle formation, account for the decrease in cuticle deposition during exposure to surface moisture. These included genes involved in the synthesis of monomers and constituents (*GPAT6*, *KCS10*, *SHN3*, *WSD1*, *CER6*) and their transport across the plasma membrane (*ABCG11*). The down-regulation occurred at the same time as microcracks formed [15,16], as cuticle thickness around microcracks decreased [16] and as the amounts of the amounts of the major constituents of cutin and wax decreased. These observations suggest a causal relation between moisture exposure, a reduction in the expressions of genes involved in cuticle synthesis, a decrease in cuticle mass and the subsequent formation of a periderm and the onset of suberin synthesis and deposition. Because moisture and its removal affected all levels of potential control (synthesis, transport and transcriptional regulation) it is most plausible that these associations are causal, rather than merely correlative.

#### 3.1.1. Cutin, Wax and Suberin Synthesis

Moisture exposure during Phase I, down-regulated *GPAT6*. *GPAT6* and its orthologs have important functions in cuticle formation for example in the synthesis of 2-monoacylglycerols as shown for *Arabidopsis* [23]. A defect of an orthologous gene in tomato *SlGPAT6* led to reduced cutin content and decreased cuticle thickness compared to the wildtype [24]. Consistent with this is the observation by Legay [9] who reported decreased gene expression of *MdGPAT6* in russeted as compared to non-russeted apple cultivars. This is in line with our observation of decreased expression of *GPAT6* during moisture exposure.

Decreased expression during moisture exposure was also observed for *KCS10* in russeted fruit skins [9]. *KCS10* is involved in long-chain fatty acid synthesis in *Arabidopsis* [25]. Furthermore, Legay [9] also observed a down-regulation of genes involved in the synthesis of wax constituents such as *WSD1* and *CER6* in skins of russeted fruit. In *Arabidopsis*, *WSD1* is involved in the synthesis of wax esters. It also has diacylglycerol acyltransferase activity [26]. *CER6* is involved in the elongation of C_24_ very long chain fatty acids (VLCFAs). A loss of function in *Arabidopsis* led to an accumulation of the C_24_ wax component [27].

After moisture removal, genes related to the synthesis of suberin and, possibly, the formation of a periderm (*ABCG20*, *CYP86B1*, *MYB93*, *MYB42*, *NAC038* and *NAC058*) were subsequently up-regulated. This is consistent with an up-regulation of the expressions of *CYP86B1*, *MYB93*, *NAC038* and *NAC058* in skins of russeted apple fruit, but not in non-russeted mature fruit [9]. In *Arabidopsis*, *CYP86B1* is involved in the synthesis of ω-hydroxy-C_22_ and -C_24_ acids and α,ω-dicarboxylic acids. A knockout of this gene led to an accumulation of C_22_ and C_24_ fatty acids [28]. For *NAC038* and *NAC058* an involvement in the synthesis of suberin monomers is not unlikely. Experiments on overexpression of *MdMyb93* in *N*. *benthamiana* not only led to an increased suberin formation but also to an upregulation of *NAC038* and *NAC058* orthologues of *Nicotiana* [21].

#### 3.1.2. Transport of Cutin Monomers, Wax Constituents and Suberin Monomers

During moisture exposure (Phase I) genes involved in the transport of cutin monomers or wax constituents across the plasma membrane were down-regulated. These included *ABCG11* that encodes an ATP binding cassette transporter essential for the transport of cuticular lipids in *Arabidopsis* (*AtABCG11*; [29]). The related orthologous gene *MdABCG11* (MDP0000200335) of apple was localized in a major QTL controlling russeting of ‘Renetta Grigia di Torriana‘ [7]. Also, *MdABCG11* was down-regulated in russeted as compared to non-russeted cultivars in a bulk transcriptomic study [9].

The ABCG transporters *ABCG2*, *ABCG6* and *ABCG20* are involved in the transport of suberin monomers in *Arabidopsis* [30]. The up-regulation of *ABCG20* after termination of moisture exposure (Phase II) during the period of periderm formation in apple fruit skin therefore implies a requirement for transport of suberin monomers across the plasma membrane as would be needed for suberin incrustation of the phellem cell walls. At 8 d after moisture removal, ω-hydroxy-C_22_ acid had increased there and even more so at 113 d. This monomer is associated with russeted fruit skin at maturity [31].

#### 3.1.3. Transcriptional Regulation of Cutin, Wax, and Suberin Synthesis

Moisture exposure also affected the transcriptional regulation of cuticle development by *SHN3*. The SHN transcription factor genes are known as positive regulators of cuticle formation and of patterning of epidermal cells in *Arabidopsis* and tomato [32,33,34]. The silencing of *SlSHN3* in tomato led to reduced amounts of cuticular lipids and alterations in cuticle morphology [34]. In apple fruit, markers linked to the *MdSHN3* gene co-segregate with decreased cuticle thickness, increased microcracking, decreased expression in russeted clones compared to non-russeted ones and increased potential for russet formation [8].

*MYB93* is a key factor for the transcriptional regulation of suberin deposition in apple. It affects the synthesis and transport of suberin monomers, and their polymerization [21]. The transcription factor *MYB42* is involved in the synthesis of secondary cell wall, specifically in secondary cell wall thickening [35]. *MYB42* is also involved in the activation of genes for synthesis of lignin and phenylalanine, which serves as a precursor of many secondary metabolites in *Arabidopsis* [36]. We observed an up-regulation of the expression of *MYB42* during early formation of periderm whereas Legay [9] observed a down-regulation. The reason for this discrepancy is unknown. Increased expression of *MYB42* indicates concurrent lignin synthesis and secondary cell wall thickening during early phases of russeting. *NAC038* and *NAC058* also increased during Phase II of russet formation but their functions are not yet known.

### 3.2. Metabolites

The decreased expression of genes involved in cutin and wax synthesis resulted in decreased deposition in moisture-exposed skin patches. The 16-hydroxy-C_16_ acid, 10,16-dihydroxy-C_16_ acid and 9,10,18-trihydroxy-C_18_ acid are major constituents of cutin [37,38,39]. Furthermore, C_16_ acids are more abundant in the cutin of young and rapidly expanding organs and the amount of C_18_ acids increases as the organ develops and matures [38,39]. This was also observed in this study of apple fruit cutin. The mass of these three major constituents significantly decreases after 12 d moisture exposure. At maturity (113 d), the mass of the three major constituents was still lower in the moisture-exposed skin patches than in the control ones.

Within the wax fraction the C_27_ and C_29_ alkanes, the C_26_ and C_28_ primary alcohols, sterols and the triterpenes ursolic and oleanolic acid, are the dominant constituents in apple fruit wax [31,40,41,42,43]. These constituents are typical of the wax of *Rosaceae* species [44]. These constituents all decreased during moisture exposure indicating a decrease in the expressions of wax-related genes, paralleled by corresponding decreases in synthesis and deposition. Similarly, Legay [31] reported decreased masses of ursolic acid and oleanolic acid in russeted apple skins at maturity, compared to non-russeted skins.

Deposition of wax in microcracks is an effective repair mechanism that re-establishes the cuticle’s barrier function [15,45,46]. Furthermore, wax deposition in the cuticle of an expanding fruit surface converts elastic strain into plastic strain, thereby fixing both strain and stress [47]. Our observations suggest that decreased expression of genes involved in cutin and wax synthesis during moisture exposure led to decreased deposition. This may have contributed to, or even caused, the increased microcracking of the cuticle.

The increase in suberin content is less clear from the analysis of composition. First, most constituents of suberin also serve as monomers in cutin synthesis. Notable exceptions are the long chain (C_20_, C_22_, C_24_) ω-hydroxy acids that are unique for suberin [31,48]. Second, despite a marked and consistent up-regulation of genes involved in synthesis of monomers for suberin, there was no clear corresponding increase in suberin monomers 8 d after discontinuation of moisture exposure. At this stage, a periderm had begun to develop in the hypodermal cell layers, in this and also our earlier study, as inferred from cross-sections of skin patches [16]. However, when skin patches were incubated in pectinase and cellulase, the cell layers separating the periderm from the epidermis were digested and, hence, the developing islands of periderm were lost to the isolation medium. This observation explains, why the periderm was detectable in cross-sections of the skin 8 d after discontinuation of moisture exposure but were not evident in the isolated cuticle polymer or as a major chemical constituent in the mass spectra of the moisture-exposed cuticles of fruit skins. By 113 d a complete periderm had developed, and this extended to the skin surface in the moisture-exposed fruit. This periderm remained attached to the cuticle during isolation at 113 d, but not at 8 d after moisture exposure was discontinued. Consequently, the characteristic constituents of suberin were clearly detectable. The slight increase in the un-exposed control patches does not conflict with the above conclusion. This suberin is accounted for by the presence of lenticels that form in the apple fruit skin during normal development.

Unfortunately, the overlap of many constituents between suberin and cutin made it impossible to calculate the amount of suberin deposited in moisture-exposed skin patches simply by summation. Further, moisture-treated skin patches are composite polymers comprising both cuticle and periderm to varying extents. For these a first estimate of the total amount of suberin present may be obtained by using pure suberin from the bark periderm of the trunk. In contrast to the moisture-treated fruit skin patches, the isolated periderm of the bark of the trunk is comprised of suberin only, there is no cuticle. Using the bark periderm of ‘Pinova’ apple trees as a reference, the masses of the suberin constituents relative to those of the three suberin-specific character constituents, i.e., the ω-hydroxy-C_20_, -C_22_ and -C_24_ acids was calculated. This analysis revealed a marked increase in suberin deposition in line with that expected, based on the increases in gene expression.

### 3.3. Russet Susceptibility is Highest during Early Fruit Development

The histological, biochemical and molecular results demonstrate that moisture-induced russet is limited to the early stages of fruit development [16]. This is consistent with field observations where the first four weeks after full bloom are considered critical [1,10,11,12,13,14]. Moisture exposure occurring later in fruit development (for example between 66 and 78 DAFB or 93 and 105 DAFB) resulted in only slight decreases in expression of cutin- and wax-related genes and no increases in expression of suberin-related genes. This is consistent with the observed lack of periderm formation [16] and the lack of visual symptoms of russeting [15]. The higher susceptibility to russet during early fruit development results from the high relative area growth rates at this stage [49]. Unless matched by high rates of cutin and wax deposition [17], high relative area growth rates (high rates of strain) result in microcracking. Thus, growth strain, microcracking, macrocracking and russeting are interrelated [4,46,50,51,52].

### 3.4. Conclusion

**Figure 10:**
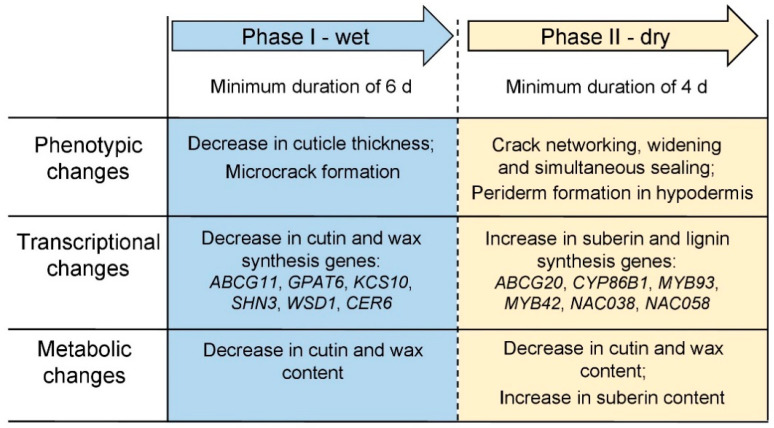
Schematic of the process of russeting at the phenotypic, transcriptional and metabolic level during exposure of apple fruit skin patches to moisture (Phase I) and following discontinuation of exposure (Phase II).

The molecular and biochemical results presented here are consistent with the histological observations reported earlier [16]. Based on both studies, russeting must be viewed as a two-step process comprising the following sequence of events (Figure 10). A young fruit, that typically has a high growth rate and, hence, a strain rate of the skin [17], responds to surface moisture by decreasing cutin and wax synthesis and deposition due to the down-regulation of *ABCG11*, *GPAT6*, *KCS10*, *SHN3*, *WSD1* and *CER6*. As a consequence, the fixation of elastic strain by cutin and wax deposition is decreased and so, elastic strain builds up [47]. The increase in strain and (possibly) a change in the rheological properties of the cuticular membrane (CM) due to hydration [53] results in the formation of microcracks. These microcracks generally extend tangentially and so form a crack network on the fruit surface that continues to extend even after moisture exposure is discontinued. As a result, the cuticle’s barrier function is impaired. A deposition of wax in developing microcracks may ‘repair’ the microcrack and so restore the cuticle’s barrier function [45,54,55] and so avert the development of russeting. However, if this repair process lags too far behind, Phase II of the russeting cascade is initiated [46]. Following drying of the fruit surface, a yet unknown signal triggers the formation of a periderm. This signal must be transmitted from the microcrack (or the immediate vicinity thereof) deeper down to the hypodermal cell layers where a periderm begins to differentiate. Genes involved in suberin and lignin synthesis including *ABCG20*, *CYP86B1*, *MYB93*, *MYB42*, *NAC038* and *NAC058* are all up-regulated. Suberin is deposited in the cell walls of the phellem. The process continues until the cuticle and epidermis and the outer hypodermis dry and are sloughed off and the phellem becomes exposed at the skin surface. The suberized phellem now appears as the typical rough, dull brown of a russeted fruit skin. 

The triggers have not yet been identified that lead to the differential expression of both cutin- and wax-related genes during moisture exposure, nor those of the suberin-related genes after moisture exposure is discontinued. It is speculated that the expression of suberin-related genes is triggered by the impaired barrier properties of the cuticle. Potential candidates for this trigger are a high (O_2_), a low (CO_2_) or a more negative water potential in the tissues immediately subtending a microcrack. Interestingly, in potato, a low (O_2_) inhibited suberization of the tuber following wounding [56]. Further experiments employing techniques such as transcriptomic analysis would be helpful in identifying the potential triggers for the down-regulation of expression of the genes associated with cutin and wax synthesis during moisture exposure, as well as for the up-regulation of suberin-synthesis genes after moisture exposure has been discontinued.

The model of periderm formation presented here will apply equally to other fruitcrop species that develop microcracks in the cuticle during the early phase of development and subsequently russeting (e.g., pear). However, fruitcrop species that bear fleshy fruit and that are susceptible to cracking, usually are not susceptible to russet. In these, a comparable mechanism for fixing the impaired barrier properties of the fruit skin at that stage of development is absent. 

## 4. Materials and Methods

### 4.1. Plant Materials

‘Pinova’ apple trees (*Malus × domestica*, Borkh.) grafted on M9 rootstocks were cultivated in the experimental orchards of the horticultural research station of the Leibniz University Hanover at Ruthe (52°14′ N, 9°49′ E) according to current regulations for integrated fruit production. Developing fruit were sampled randomly over three growing seasons from a total of 125 trees. For comparison, bark sections were excised from the base of the trunks of 21-year-old ‘Pinova’ trees about 10 cm above the graft union.

### 4.2. Moisture Treatment

Flowering spurs were randomly selected, and the clusters thinned at full bloom to one flower per cluster—usually the king flower. The moisture treatments were started when fruits had reached 10–12 mm diameter (usually about 21–31 DAFB). Experiments were carried out in two consecutive Phases. During Phase I a skin patch was exposed to moisture. For the subsequent Phase II, exposure to moisture was discontinued. For the moisture treatment, a 2 mL polyethylene tube (8 mm diameter; Eppendorf, Hamburg, Germany) was cut to 17 mm length and a hole (1.4 mm diameter) drilled into the tip. The tube was fixed in the equatorial plane of the fruit using a non-phytotoxic silicone rubber (Dowsil™ SE 9186 Clear Sealant; Dow Toray, Japan) [15]. Following curing, the tube was filled through the hole in the tip with deionized water using a syringe. The hole in the tip was then sealed with silicone rubber. The silicone was inspected for leakage and resealed every second day. The opposite, un-treated side of the same fruit served as the control [15]. The duration of moisture exposure (Phase I) was either 6 or 12 d. Thereafter, the tube was carefully removed. Unless specified otherwise, formation of a periderm was monitored up to 113 d after termination of the moisture treatment (Phase II).

### 4.3. RNA Extraction

Apple fruit skin from moisture-exposed and unexposed (control) areas were excised using a razor blade and immediately frozen in liquid N_2_. Fruit skins were stored at −80 °C till processing. The skin tissue was ground to a powder with pestle and mortar in liquid N_2_. RNA extraction was done using the InviTrap Spin Plant RNA Mini Kit (STRATEC Molecular GmbH, Berlin, Germany) according to the manufacturer’s protocol. To remove genomic DNA, total RNA was treated with DNase using the DNA-free^TM^ Kit (Thermo Fisher Scientific, Waltham, MA, USA). RNA purity and quantity were determined by measuring the absorbance at 230, 260 and 280 nm using a Nanodrop 2000c spectrophotometer (Thermo Fisher Scientific, Waltham, MA, USA). RNA integrity was determined on a 1.5% agarose gel. cDNA synthesis was carried out with the LunaScript^®^ RT SuperMix Kit (New England Biolabs, Ipswich, MA, USA) using 600 ng of RNA in a 40 µL reaction volume following the manufacturer′s protocol. The number of biological replicates was from three to five. Each biological replicate comprised the skin from six to ten fruits.

### 4.4. Quantitative Real-Time PCR

Gene expression was determined by quantitative real-time PCR using the QuantStudio™ 6 Flex Real-Time PCR System (Applied Biosystems, Waltham, MA, USA). Genes observed in this study are listed in Table 1 and the corresponding specific primers in Table S1. Primer design was done using the Primer3 software (Primer3, http://primer3.ut.ee/). Gene expression values each represent three to five biological replicates and two to three technical replicates. To normalize gene expression, the reference genes *PROTEIN DISULFIDE ISOMERASE* (*PDI*) (MDP0000233444) and *MdeF-1alpha* (AJ223969.1) were used. Reactions were carried out using 1 µL undiluted cDNA in 8 µL volume of the Luna^®^ Universal qPCR Master Mix (New England Biolabs, Ipswich, MA, USA) following manufacture’s guidelines. The final concentration was 200 nM for each specific primer. PCR cycle conditions were: one cycle of 95 °C for 60 s, 40 cycles of 95 °C for 15 s and 60 °C for 60 s. After amplification melting curve analysis (95 °C for 15 s, 60 °C for 60 s, 60 to 95 °C in 0.5 °C increments) was used. Primer efficiency was determined in a five-fold dilution series of a cDNA pool covering five dilution points, each using the QuantStudio™ Real-Time PCR Software v1.3 (Applied Biosystems, Waltham, MA, USA). 

Relative gene expression was calculated according to Pfaffl [57]. Modifications were according to Chen [58].

### 4.5. Isolation of Fruit Cuticular Membranes and Periderm Membranes and Bark Periderm Membrane

Cuticular membranes and periderm membranes (PM) of developing apple fruit and periderm membranes from the bark (BP) of the trunk were isolated enzymatically [59]. Moisture exposed and unexposed skin samples were excised using biopsy punches (8 mm diameter, Kai Europe, Solingen, Germany; or 10 or 12 mm diameter, Acuderm, Terrace, FL, USA). The sections of the trunk bark were excised using a scalpel. Skin discs or bark sections were incubated at room temperature in 50 mM citric acid buffer at pH 4.0 containing pectinase (90 mL L^−1^; Panzym Super E flüssig, Novozymes A/S, Krogshoejvej, Bagsvaerd, Denmark), cellulase (5 mL L^−1^; Cellubrix L; Novozymes A/S) and 30 mM NaN_3_ [59]. The enzyme solution was periodically replaced until CMs and PMs separated from their adhering cellular debris. Isolated CMs, PMs and BPs were rinsed in deionized water, dried at 40 °C for 20 h and stored at room temperature.

### 4.6. Cross-Sections of Skin Segments and Isolated Cuticular Membranes/Periderm Membranes and Microscopy

Tissue blocks were cut from moisture exposed and unexposed control patches of the fruit skin, transferred into Karnovsky fixative [60] and stored at 4 °C. The blocks were rinsed in distilled water, transferred to 70% (*v*/*v*) aqueous ethanol (EtOH) for 16 h and dehydrated in an increasing series of aqueous EtOH solutions (80%, 90% and 96% EtOH (*v*/*v*) for 30 min each). Subsequently, blocks were transferred to 100% isopropanol (twice for 40 min each) and then in a xylene substitute (AppiClear AppliChem, Münster, Germany; twice for 40 min each). The dehydrated blocks were infiltrated with a 1:1 (*v*:*v*) paraffin/xylene-substitute mixture (Carl Roth, Karlsruhe, Germany) for 40 min once, followed by two infiltrations with pure paraffin for 40 min each supported by a mild vacuum (absolute pressure 10.8 kPa).The embedded ES were stored at 4 °C. Sections of 10 µm thickness were cut using a rotatory microtome (Hydrax M 55, Zeiss, Oberkochen, Germany), collected on glass microscope slides and dried for 16 h at 37 °C. The paraffin was removed using xylene substitute (twice for 10 min each). Sections were rehydrated in a decreasing series of aqueous EtOH (96%, 80%, 70%, and 60%, all for 10 min each), followed by two final incubations in distilled water for of 5 min each. Cross-sections of isolated CM or PM were obtained by hand, using a razor blade.

Sections were stained for 1 h with 0.005% Fluorol Yellow 088 (Santa Cruz Biotechnology, TX, USA) [61] dissolved in a 1:1 mixture (*v*:*v*) of melted polyethylene glycol 4000 (SERVA Electrophoresis, Heidelberg, Germany) and 90% glycerol. Sections were inspected under incident fluorescent light (filter U-MWB, 450–480 nm excitation; ≥520 nm emission wavelength) using a fluorescence microscope (BX-60, Olympus, Hamburg, Germany). Three biological replicates each were observed for the ES, CM and PM. 

### 4.7. Quantification of Wax Constituents by GC/FID and GC/MS

Isolated CM/PM discs and BP sections were cut into small pieces. An equal number of CM/PM pieces from five individual CM/PM discs (each represents a fruit) or of BP from samples of the trunk were pooled to make about 0.5 to 1 mg of material which represents a sample/replication. Samples were extracted in 5 mL chloroform overnight at room temperature on a horizontal rolling bench (CAT RM. 5–30 V, Staufen, Germany). The wax extract was immediately spiked with an adequate amount of internal standard (100 µL tetracosane of a chloroform solution of 10 mg tetracosane in 50 mL) later enabling the quantification of the single wax compounds. The chloroform volume was reduced under a gentle stream of N_2_ at 60 °C in a heating block. The extracted CM, PM and BP pieces were dried on Teflon discs for further cutin/suberin analysis. Since some wax molecules contain polar hydroxyl- and carboxyl groups which negatively interfere with the GC column, all samples were derivatized by silylation yielding the corresponding trimethylsilyl ethers and -esters. For sylilation 20 µL of BSTFA (N, O-bis(trimethylsilyl)-trifluoracetamid, Machery-Nagel, Düren, Germany) and 20 µL of pyridine (Sigma Aldrich, Deisenhofen, Germany) were added to each sample. Derivatization took place for 45 min at 70 °C in a heating block. Of each sample 1 µL was injected on-column to a gas chromatograph coupled to a flame ionization detector (GC-FID; CG-Hewlett Packard 5890 series H, Hewlett-Packard, Palo Alto, CA, USA, 307 column-type: 30 m DB-1 i.d. 0.32 mm, film 0.2 μm; J&W Scientific, Folsom, CA, USA). For identification of wax constituents, the extracted wax was analyzed by GC-MS (Quadrupole mass selective detector HP 5971, Hewlett-Packard, Palo Alto, CA, USA) by injecting 1 µL on-column. The constituents were quantified using the internal standard. Identification of the molecules was carried out by comparing fragmentation patterns with literature data and with our own data library. Data are expressed as mass per unit fruit surface area or trunk surface area. The number of replications was two to three, where each replicate comprised a subsample of five pooled CM, PM discs from five different fruit. The number of replications for the BP was three, each representing a different tree.

### 4.8. Quantification of Apple Cutin and Suberin Monomers by GC/FID and GC/MS

The extracted and dried CM/PM and BP were transesterified in glass vials by incubation in 1 mL boron trifluoride-methanol solution (BF_3_/MeOH) for 16 h at 70 °C. After cooling of the samples, 20 µg of internal standard (100 µL dotriacontane of a chloroform solution of 10 mg dotriacontane in 50 mL) was added to each sample. Saturated NaHCO_3_ (2 mL) was added to stop the depolymerization reaction. Cutin/suberin monomers were extracted three times by adding 2 mL chloroform. The chloroform phase was collected, washed by adding 1 mL HPLC grade water and then dried using NaSO_4_. The water phase was discarded. The chloroform solution containing the cutin/suberin monomers was concentrated under a gentle stream of N_2_ at 60 °C. Samples were derivatized as described above by adding 20 µL of BSTFA and 20 µL of pyridine. Monomers were quantified by injecting 1 µL of each sample on-column on a gas chromatograph coupled to a FID (GC-FID; CG-Hewlett Packard 5890 series H, Hewlett-Packard, Palo Alto, CA, USA, 307 column-type: 30 m DB-1 i.d. 0.32 mm, film 0.2 μm; J&W Scientific, Folsom, CA, USA). The individual constituents were identified on a gas chromatograph coupled to a mass spectrometer (Quadrupole mass selective detector HP 5971, Hewlett-Packard, Palo Alto, CA, USA) relative to the internal standard in each sample. Monomers were identified by comparing the fragmentation patterns with known standards from the literature or from our own library. Data are expressed as mass per unit fruit surface area. The number of replications was two or three, where each replicate comprised pooled CM/PM from five individual CM/PM discs obtained from five different fruit. The number of replications for the BP was three, each representing a different tree.

### 4.9. Data Analyses

Because moisture exposure of a patch of fruit skin results in formation of a periderm only in parts of the moisture treated area, the polymer obtained following enzymatic isolation from such surfaces is a mixed polymer comprising cuticle (cutin and wax) and periderm (suberized phellem and wax) of varying amounts. Furthermore, cutin and suberin and their waxes share common monomers and constituents. This makes it impossible to quantify the amounts of cutin and suberin or the amounts of cuticular and periderm wax deposited per unit surface area of moisture-treated patches of fruit skin. However, for the dewaxed suberin fraction, the ω-hydroxy-C_20_, -C_22_ and -C_24_ acids are major and unique constituents of suberin that together account for 17.6% of a pure suberin of bark periderm of apple tree. As a first approximation, we assumed the composition of the suberin of a composite cuticle with periderm of moisture-treated apple fruit skin and that of the bark of a trunk of the same apple cultivar to be identical. Hence, the total amount of suberin in the cuticle may be calculated relative to the amounts of the ω-hydroxy-C_20_, -C_22_ and -C_24_ acids. In contrast to the cuticle, with periderm of the moisture-treated fruit surface, the bark periderm of a trunk is comprised of suberin only—a cuticle is absent. We therefore used the suberin of the bark periderm as a standard. The periderm from the bark of the trunk were extracted, depolymerized and analyzed by GC-MS. Using the three hydroxy acids, a normalized suberin composition of the moisture-treated fruit was then calculated. This procedure allowed quantification of the time course of cutin and suberin deposition of the mixed polymer of a moisture-treated fruit surface. Due to the lack of unique constituents, the same calculation could not be carried out for the wax of cuticles with periderm.

Data are presented as means ± standard errors. When error bars are not shown, they were smaller than the data symbols. Paired sample Student’s *t*-tests were run. Significant differences between dry/dry and wet/dry at *p* ≤ 0.05 is indicated by ‘*’.

## Figures and Tables

**Table 1:** List of genes analyzed in the gene expression study.

Gene Name	Accession	AGI Locus Code	Description	Reference
**Cuticle-related**				
*ABCG11*	MDP0000200335	AT1G17840.1	ABCG11, white-brown complex homolog protein 11, cuticular lipid transport to the extracellular matrix	[29]
*CER6*	MDP0000392495	AT1G68530.1	3-Ketoacyl-CoA synthase 6, involved in the synthesis of VLCFAs	[27]
*FDH, KCS10*	MDP0000235280	AT2G26250.1	FIDDLEHEAD,3-Ketoacyl-CoA synthase 10, probably involved in synthesis of long-chain lipids	[25]
*GPAT6*	MDP0000479163	AT2G38110.1	Glycerol-3-phosphate acyl transferase 6, synthesis of cutin monomers	[24]
*SHN3*	MDP0000178263	AT5G25390	Positive transcriptional regulator of cuticle synthesis	[32]
*WSD1*	MDP0000701887	AT5G37300.1	Wax Ester Synthase/Acyl-Coenzyme A:Diacylglycerol Acyltransferase, Wax ester synthesis and diacylglycerol acyltransfer	[26]
**Periderm-related**				
*ABCG20*	MDP0000265619	AT3G53510	ATP-binding cassette G20, involved in transport of aliphatic suberin polymer precursors	[30]
*CYP86B1*	MDP0000306273	AT5G23190.1	Cytochrome P450, family 86, subfamily B, polypetide 1, synthesis of very long chain ω-hydroxyacid and α,ω-dicarboxylic acid in suberin polyester	[28]
*MYB42*	MDP0000787808	AT4G12350.1	MYB domain protein 42, involved in secondary cell wall biosynthesis and regulation of lignin synthesis	[35,36]
*MYB93*	MDP0000320772	AT1G34670.1	MYB domain protein 93, positive regulator of suberin synthesis	[21]
*NAC038*	MDP0000232008	AT2G24430.1	NAC domain containing protein 38	uncharacterized
*NAC058*	MDP0000130785	AT3G18400.1	NAC domain containing protein 58	uncharacterized

## Data Availability

Original data is available upon request from the corresponding author.

## Supplementary Materials

The following are available online at https://www.mdpi.com/2223-7747/10/1/65/s1, Figure S1: Time course of expressions of genes related to cutin, wax (a–f) and suberin and lignin synthesis (g–l) of apple fruit skin during exposure to moisture (Phase I) and after exposure to moisture ceased (Phase II). Figure S2: Time course of expressions of genes related to cutin, wax (a–f) and suberin and lignin synthesis (g–l) of apple fruit skin during exposure to moisture (Phase I) and after exposure to moisture was discontinued (Phase II). Table S1 Primers for genes analyzed used in the present study [62], Table S2: Effect of moisture exposure on the composition of the cuticle/periderm polymer.
