# Supplementary material for: Russeting in Apple is Initiated after Exposure to Moisture Ends: Molecular and Biochemical Evidence
Source: Plants (Basel). 2020 Dec 30;10(1):65. doi: 10.3390/plants10010065 (PMC7824318; doi:10.3390/plants10010065)
Supplement: Supplementary file 1 [file plants-10-00065-s001.pdf]

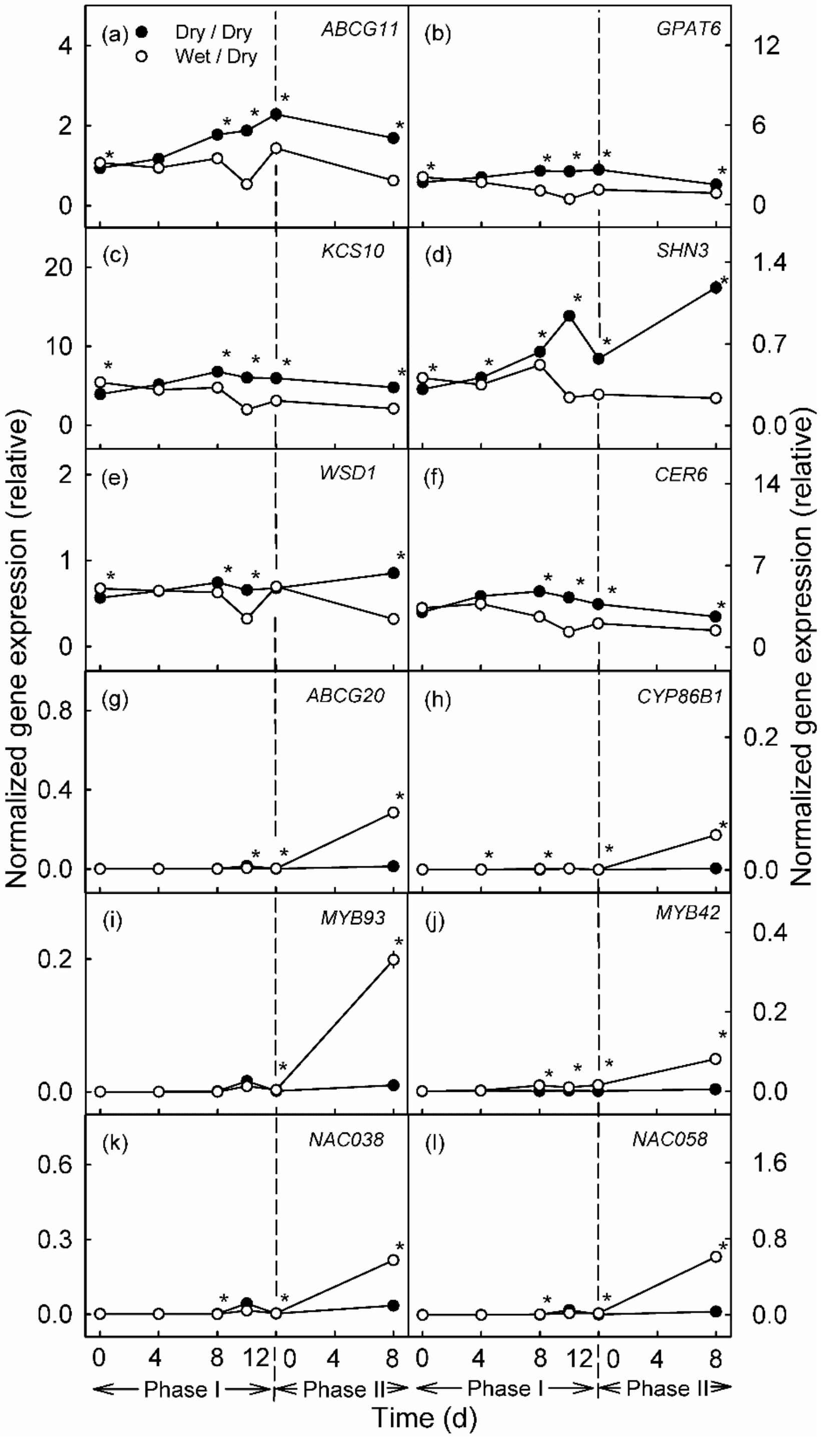

Figure S1. Time course of expressions of genes related to cutin, wax (a-f) and suberin and lignin synthesis (g-l) of apple fruit skin during exposure to moisture (Phase I) and after exposure to moisture ceased (Phase II). During Phase I, a patch of the fruit skin was exposed to moisture for 12 d beginning at 23 days after full bloom (DAFB) (wet). During the subsequent Phase II, moisture exposure was discontinued and the patch was exposed to the atmosphere (Dry). Moisture exposed patches of fruit skin are referred to as wet/dry, unexposed (control) patches as dry/dry. The end of the period of moisture exposure is indicated by the vertical dashed line. The expression values are means ± SE of three independent biological replicates comprising six fruit each. The '*' indicates significant difference between dry/dry and wet/dry at $p \leq 0.05$ (Student's t-test).

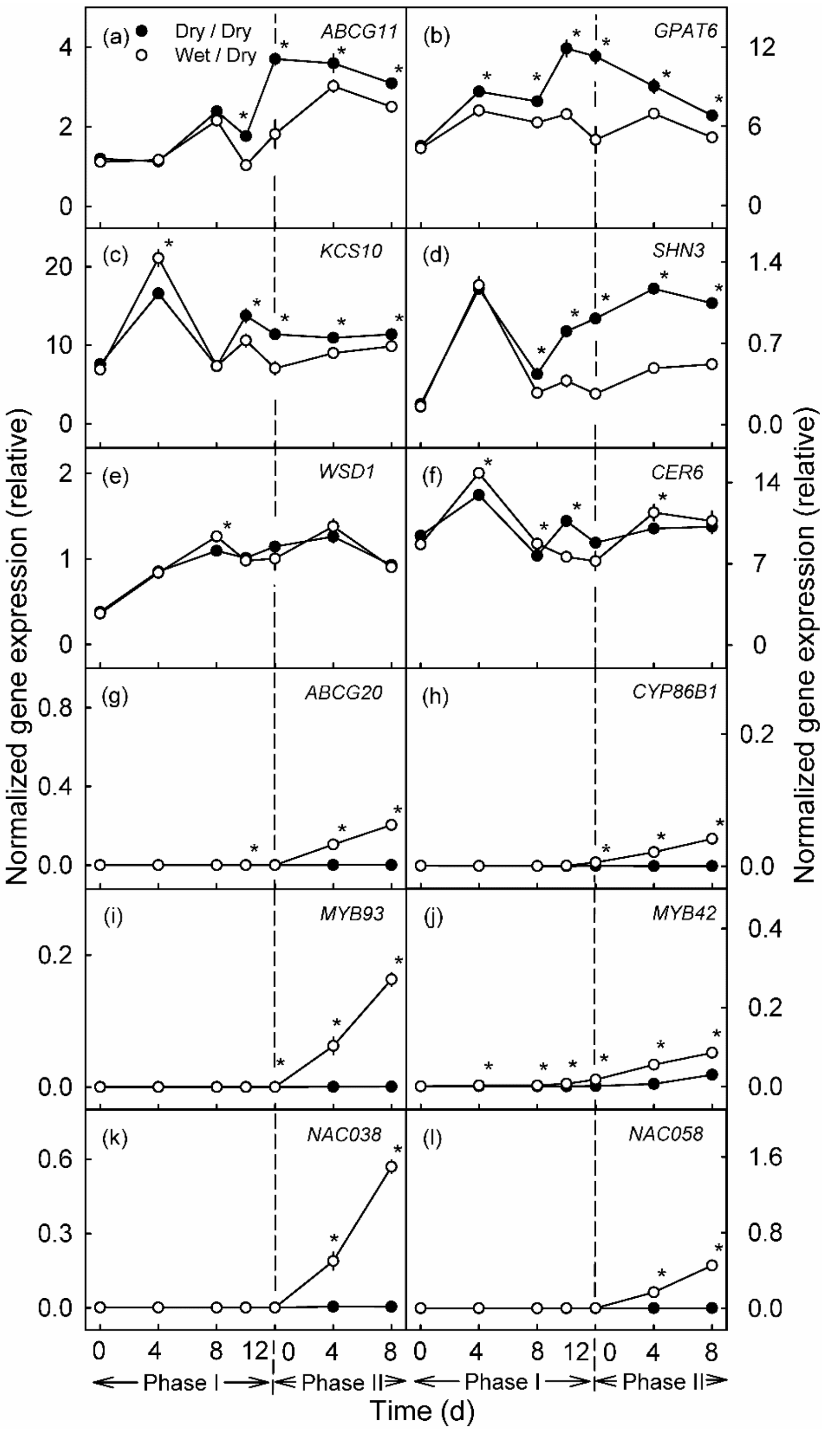

Figure S2. Time course of expressions of genes related to cutin, wax (a-f) and suberin and lignin synthesis (g-l) of apple fruit skin during exposure to moisture (Phase I) and after exposure to moisture was discontinued (Phase II). During Phase I, a patch of the fruit skin was exposed to moisture for 12 d beginning at 21 days after full bloom (DAFB) (wet). During the subsequent Phase II, moisture exposure was discontinued and the patch exposed to the atmosphere (Dry). Moisture exposed patches of fruit skin are referred to as wet/dry, unexposed (control) patches as dry/dry. The end of the period of moisture exposure is indicated by the vertical dashed line. The expression values are means ± SE of three independent biological replicates comprising six fruit each. The '*' indicates significant difference between dry/dry and wet/dry at $p \leq 0.05$ (Student's t-test)

Table S1. Primers for genes analyzed used in the present study.

Gene name	Accession	Primer sequence (5'-3') Forward Primer	Reverse Primer	PCR efficiency (%)	Reference
ABCG11	MDP0000200335	TGGCGGGTTTCCTTCTTTCA	CACAAATGCAGTAACGCCGT	98.7	This study
ABCG20	MDP0000265619	ACTGGGCATGGACAACAACA	ATTTTCCCGACCCACTTGCT	102.9	This study
CER6	MDP0000392495	AGCAACAACCCTAAGAGCGT	GTTGGGCGGAATGTAGTGGA	85.0	This study
CYP86B1	MDP0000306273	CGCTTTGTGACCCCATCC	AATGACGTCTTCCGCAAACT	109.3	[9]
eF-1alpha	AJ223969.1	ACTGTTCCTGTTGGACGTGTTG	TGGAGTTGGAAGCAACGTACCC	93.0	[9]
GPAT6	MDP0000479163	TCTTGAACCAGCTACCGTCG	AATCCCAAAGTCCCAGCCAA	91.0	This study
KCS10	MDP0000235280	TGCTGAGGTGGGAAGTTTGA	ACACCAAAGAACCCTAGCACA	91.6	This study
MYB42	MDP0000787808	CCTTGGCAATAGGTGGTCGA	TGATGTGCGTGTTCCAGTGA	94.2	This study
MYB93	MDP0000320772	TGGACAAACTATCTTAGGCCGG	GTTGCCGAGGATGGAATGGA	102.5	This study
NAC038	MDP0000232008	CGGCGGATCATCAAGTAGCA	AAACCCTCCTCCTCCTCCAA	84.6	This study
NAC058	MDP0000130785	AGCCACAACAAGCAACAACA	TTTGGCAGCTCGATGTCTCC	90.7	This study
PDI	MDP0000233444	TGCTGTACACAGCCAACGAT	CATCTTTAGCGGCGTTATCC	100.6	[62]
SHN3	MDP0000178263	GGGACGTTTGAGACAGCAGA	TTTTGGTCGGTGGCAGGTTT	93.6	This study
WSD1	MDP0000701887	AGAAATGGTCAAACCCGACA	AGACGAAGTCAAGCGCATTT	91.1	[9]

Table S2. Effect of moisture exposure on the composition of the cuticle/periderm polymer. During exposure to moisture and following discontinuation of exposure a mixed polymer comprising cutin and suberin formed. Using the bark periderm of the trunk (BP) of 'Pinova' apple trees, the composition of pure suberin was determined. Assuming the suberin composition of periderms of terrestrial surfaces within the same species and cultivar to be identical, the content of ω-hydroxy-C_{20}, -C_{22}, and -C_{24} acids in bark suberin was used to calculate a normalized suberin composition and mass per unit area for the mixed cuticle/periderm surface of the fruit. The experiment was run as a two-phase experiment. During Phase I, the fruit were exposed to moisture for 12 d beginning at 31 days after full bloom (DAFB) ('wet'). Moisture exposure was discontinued at the onset of Phase II ('dry'). The treated patches of fruit skin were sampled 113 d after moisture exposure ceased. Patches of fruit skin that remained dry during both phases served as control (dry/dry). For details of the analysis see Materials and Methods. The study was carried out using three replicates. ND = not detected.

	Fruit Cutin and Suberin (μg cm^{-2})		Bark Suberin (μg cm^{-2})	Bark Suberin (%)	Fruit Cutin (μg cm^{-2})		Fruit Suberin (μg cm^{-2})	
	Dry/Dry	Wet/dry			Dry/Dry	Wet/Dry	Dry/Dry	Wet/Dry
Aromatics								
Hydrocinnamic acid	11.8 ± 3.3	7.4 ± 1.7	ND	ND	11.8 ± 3.3	7.4 ± 1.7	0.0 ± 0.0	0.0 ± 0.0
Cinnamic acid	ND	ND	5.0 ± 1.0	0.5	ND	ND	ND	ND
cis-Coumaric acid	1.1 ± 0.3	0.7 ± 0.1	ND	ND	1.1 ± 0.3	0.7 ± 0.1	0.0 ± 0.0	0.0 ± 0.0
trans-Coumaric acid	6.3 ± 1.4	1.7 ± 0.6	ND	ND	6.3 ± 1.4	1.7 ±0.6	0.0 ± 0.0	0.0 ± 0.0
Benzoic acid 1	ND	ND	2.9 ± 0.6	0.3	ND	ND	ND	ND
Benzoic acid 1	ND	ND	4.8 ± 1.4	0.5	ND	ND	ND	ND
trans-Ferulic acid	ND	ND	39.3 ± 4.4	4.0	ND	ND	ND	ND
Linoleic acid	ND	ND	10.4 ± 1.5	1.1	ND	ND	ND	ND
Carboxylic acids								
C16	8.0 ± 1.2	6.3 ± 0.8	4.6 ± 0.9	0.5	8.0 ± 1.2	6.3 ± 0.8	0.0 ± 0.0	0.0 ± 0.0
C18	5.1 ± 0.8	3.2 ± 0.3	5.5 ± 1.9	0.6	5.1 ± 0.8	3.2 ± 0.3	0.0 ± 0.0	0.0 ± 0.0
C20	1.2 ± 0.5	0.2 ± 0.1	8.3 ± 0.7	0.8	1.2 ± 0.5	0.2 ± 0.1	0.0 ± 0.0	0.0 ± 0.0
C22	1.2 ± 0.2	16.4 ± 4.1	80.6 ± 5.3	8.2	1.2 ± 0.2	15.4 ± 3.7	0.0 ± 0.0	1.0 ± 0.4
C24	4.7 ± 2.7	1.1 ± 0.2	138.1 ± 17.5	14.0	4.6 ± 2.7	1.0 ± 0.2	0.1 ± 0.0	0.1 ± 0.0
C26	0.9 ± 0.4	2.9 ± 0.8	16.6 ± 0.9	1.7	0.9 ± 0.4	2.9 ± 0.8	0.0 ± 0.0	0.0 ± 0.0
Primary alcohols								
C16	ND	ND	1.7 ± 0.4	0.2	ND	ND	ND	ND
C18	ND	ND	1.4 ± 0.6	0.1	ND	ND	ND	ND
C20	ND	ND	1.9 ± 0.2	0.2	ND	ND	ND	ND
C22	ND	ND	34.8 ± 2.9	3.5	ND	ND	ND	ND
C24	ND	ND	39.4 ± 19.3	4.0	ND	ND	ND	ND
C26	5.5 ± 0.3	1.2 ± 0.4	62.5 ± 7.3	6.3	5.5 ± 0.4	1.1 ± 0.4	0.0 ± 0.0	0.0 ± 0.0
C28	0.4 ± 0.1	0.2 ± 0.0	15.5 ± 2.2	1.6	0.4 ± 0.1	0.2 ± 0.0	0.0 ± 0.0	0.0 ± 0.0

Table S2. Continued

	Fruit cutin and Suberin (μg cm^{-2})		Bark suberin (μg cm^{-2})	Bark Suberin (%)	Fruit Cutin (μg cm^{-2})		Fruit Suberin (μg cm^{-2})	
	Dry/Dry	Wet/Dry			Dry/Dry	Wet/Dry	Dry/Dry	Wet/Dry
Dicarboxylic acids								
C16	2.9 ± 0.3	8.7 ± 1.5	26.5 ± 3.1	2.7	2.9 ± 0.3	8.5 ± 1.5	0.0 ± 0.0	0.2 ± 0.1
C16:9,10-dihydroxy	7.3 ± 2.1	8.2 ± 0.5	ND	ND	7.3 ± 2.1	8.2 ± 0.5	0.0 ± 0.0	0.0 ± 0.0
C18	ND	ND	27.9 ± 3.1	2.8	ND	ND	ND	ND
C18:1-hydroxy	ND	ND	53.6 ± 7.8	5.4	ND	ND	ND	ND
C18:9,10-dihydroxy	7.6 ± 0.5	7.2 ± 1.7	ND	ND	7.6 ± 0.5	7.2 ± 1.7	0.0 ± 0.0	0.0 ± 0.0
C20	ND	ND	16.8 ± 8.7	1.7	ND	ND	ND	ND
C22	ND	ND	7.6 ± 0.9	0.8	ND	ND	ND	ND
Hydroxy acids								
C16:ω	28.2 ± 2.3	9.3 ± 2.3	16.3 ± 2.2	1.7	28.2 ± 2.3	9.2 ± 2.3	0.0 ± 0.0	0.1 ± 0.0
C18:ω	ND	ND	22.3 ± 2.6	2.3	ND	ND	ND	ND
C18:1	17.9 ± 3.3	27.6 ± 7.6	136.0 ± 13.8	13.8	17.8 ± 3.3	25.0 ± 6.8	0.1 ± 0.0	2.7 ± 1.0
C18:2	0.8 ± 0.3	0.4 ± 0.0	ND	ND	0.8 ± 0.3	0.4 ± 0.0	0.0 ± 0.0	0.0 ± 0.0
C20:ω	3.3 ± 0.7	5.9 ± 2.0	39.9 ± 4.2	4.0	0.0 ± 0.0	0.0 ± 0.0	3.3 ± 0.7	5.9 ± 2.0
C22:ω	1.0 ± 0.4	18.7 ± 4.7	88.2 ± 8.3	9.0	0.0 ± 0.0	0.0 ± 0.0	1.0 ± 0.4	18.7 ± 4.7
C24:ω	0.3 ± 0.1	5.8 ± 1.3	45.1 ± 3.0	4.6	0.0 ± 0.0	0.0 ± 0.0	0.3 ± 0.1	5.8 ± 1.3
C16:10,16-di	192.1 ± 20.2	46.2 ± 18.9	ND	ND	192.1 ± 20.2	46.2 ± 18.9	0.0 ± 0.0	0.0 ± 0.0
C18:9,10,18-tri	58.1 ± 14.3	53.8 ± 14.7	ND	ND	58.1 ± 14.3	53.8 ± 14.7	0.0 ± 0.0	0.0 ± 0.0
HA1	85.8 ± 24.0	32.8 ± 8.3	ND	ND	85.8 ± 24.0	32.8 ± 8.3	0.0 ± 0.0	0.0 ± 0.0
Unidentified								
X1	ND	ND	4.2 ± 2.7	0.4	ND	ND	ND	ND
X2	ND	ND	9.6 ± 7.4	1.0	ND	ND	ND	ND
X3	ND	ND	10.6 ± 2.0	1.1	ND	ND	ND	ND
X4	ND	ND	2.2 ± 0.6	0.2	ND	ND	ND	ND
X5	ND	ND	3.8 ± 1.0	0.4	ND	ND	ND	ND
X6	ND	ND	0.9 ± 0.3	0.1	ND	ND	ND	ND
Total:	451.4 ± 65.2	265.7 ± 33.1	984.8 ± 86.8	100	446.6 ± 65.3	231.3 ± 32.2	4.8 ± 0.6	34.4 ± 9.0
